# Is the clinical lipidomics a potential goldmine?

**DOI:** 10.1007/s10565-018-9441-1

**Published:** 2018-07-21

**Authors:** Linlin Zhang, Xianlin Han, Xiangdong Wang

**Affiliations:** 1 Zhongshan Hospital Institute of Clinical Science, Shanghai Institute of Clinical Bioinformatics, Fudan University Shanghai Medical School, Shanghai, China; 2 Barshop Institute for Longevity and Aging Studies, Glenn Biggs Institute for Alzheimer’s & Neurodegenerative Diseases, Department of Medicine, Division of Diabetes, Department of Biochemistry and Structural Biology, University of Texas Health Science Center at San Antonio, San Antonio, USA

**Keywords:** Clinic lipidomics, Biomarkers, Diseases, Metabolism enzymes

## Abstract

Clinical lipidomics is a new extension of lipidomics to study lipid profiles, pathways, and networks by characterizing and quantifying the complete lipid molecules in cells, biopsy, or body fluids of patients. It undoubtfully has more values if lipidomics can be integrated with the data of clinical proteomic, genomic, and phenomic profiles. A number of challenges, e.g., instability, specificity, and sensitivity, in lipidomics have to be faced and overcome before clinical application. The association of lipidomics data with gene expression and sequencing of lipid-specific proteins/enzymes should be furthermore clarified. Therefore, clinical lipidomics is expected to be more stable during handling, sensitive in response to changes, specific for diseases, efficient in data analyses, and standardized in measurements, in order to meet clinical needs. Clinical lipidomics will become a more important approach in clinical applications and will be the part of “natural” measures for early diagnosis and progress of disease. Thus, clinical lipidomics will be one of the most powerful approaches for disease-specific diagnosis and therapy, once the mystery of lipidomic profiles and metabolic enzymes is deciphered.

Clinical lipidomics is a new extension of lipidomics to study lipid profiles, pathways, and networks by characterizing and quantifying the complete spectrum of lipidomes in cells, biopsies, or body fluids of patients, and to link the lipidomics components to clinical proteomics, genomics, and phenomics (Lv et al. 2018a, b). In addition to linking new monogenic congenital lipid metabolic errors, there are an increasing evidence that lipid metabolism is associated with complex genetic characteristics in diabetes, breast cancer, Alzheimer’s disease, and lung diseases (Postle AD. Postle 2012; Han X. Han 2016; Yang K, Han X Yang and Han 2016). This indicates the potential benefit of clinical lipidomics in identification and development of disease biomarkers, exploration of signals and metabolic processes, and providing insights into molecular mechanisms and drug targets. Advances in clinical bioinformatics help us to deeply understand the increasing raw data and extracting relevant information on clinical lipidomics and to produce biological insights. A large number of methodologies has been developed in clinical lipidomics, e.g., from shotgun lipidomics, chromatographic methods containing gas chromatography and liquid chromatography and mass spectrometry imaging, to new one-phase extraction method (Yang and Han 2016; Yang L et al. Yang et al. 2016). Lipids are divided into eight categories, fatty acyls, glycerolipids, glycerophospholipids, sphingolipids, saccharolipids, polyketides, prenol lipids, and sterol lipids, of which subgroups can be further divided according to the chemical composition.

As one of the leading global projects, the LIPIDMAPS database contains more than 37, 500 unique structures of biologically related lipids, on basis of this classification (Vaz FM et al. Vaz et al. 2015). There are over1000 lipid-associated protein/enzyme genes and over 2000 proteins associated with *Homo sapiens*. It is a challenge to define specific information about lipid-associated enzymes, since they are rarely systematically collated or mined, and hard classified on basis of lipids and their corresponding metabolites. Clinical lipidomics plays the important role in prevention, diagnosis, and potential therapies, through the understanding of links between the lipids and their corresponding diseases. The identification and development of disease-specific biomarkers using clinical lipidomics require defining lipidomic profiles correlated with disease types and subtypes, severities, durations, and phases, especially with clinical phenomes (Wang X. Wang 2018; Wang X. Wang 2016; Xu M et al. Xu and Wang 2017; Zhu Z. Zhu et al. 2017). The discovery of lipid-based biomarkers can be an alternative for the diagnosis of various diseases, such as phosphatidylinositol (PI) species, PI (16:0–16:1) and PI (18:0–20:4) as potential biomarkers for breast cancer (Postle AD. Postle 2012).

To define the biological properties and functions of lipid-specific proteins/enzymes is also an important part of clinical lipidomics. Those lipid-specific proteins/enzymes can be cataloged in disease by classification and sub-classification of lipids. The synthesis and decomposition of those corresponding enzyme classification can also demonstrate lipid classification, function, or subtypes, although there are still a number of challenges to be faced and overcome. It will be important to furthermore investigate lipidomic profiles in various diseases and their corresponding metabolic enzymes, and build a network of those lipid-specific enzymes. Such a large enzyme network can help us to dynamically detect the metabolic processes of lipids, the interaction of enzyme activations in multiple pathways, and the biochemical homeostasis of cellular lipidomes. It is also questioned whether the activation of lipid-specific enzymes can be markers for lipid metabolism, in addition to the changed regulation of those enzyme expressions. The abnormal expression of some enzymes as novel targets can be the result of disease per se and target-specific therapy. For instance, the mutation of hydroxylcoenzyme A dehydrogenase alpha subunit gene which implants a key enzyme of lipid metabolism, resulting in deficiency of enzymatic activity, might contribute to the tumorigenesis of renal cell carcinoma, and other cellular processes of lipid metabolism, e.g., biosynthesis, ketogenesis, and ketolysis. Lv et al. conducted the first trial to integrate clinical lipidomics with genomic expression profiles of lipid-associated enzymes (Lv J et al. Lv et al. 2018). In this particular study, the amount of circulating lipids in patients with different subtypes of lung cancer was correlated with the expression of lipid-associated enzyme genes mined from global databases. It is definitely an outstanding innovation to clarify the specific association of lipidomic profiles with other omics data, e.g., genomics, proteomics, glycomics, and phenome, coined as clinical trans-omics (Wang X. Wang 2018).

Lipidomics has been increasingly applied to study lipid dysfunction in combination with clinical practice and to provide valuable data for the pathogenesis of many diseases. Those studies on clinical lipidomics demonstrated a very clear and consistent conclusion that there are a large number of lipid types and species in human plasma and an unexpected complexity of changes under disease conditions (Kim WS et al. Kim et al. 2018). Similar occurrence are in other body fluids, such as urine, tears, and saliva. It is another challenge to standardize the measurements of those body fluids, due to uncontrolled procedures of sampling, collecting, transporting, storing, and measuring. Lipids are extremely susceptible to oxidation processes and can be deteriorated in each process during sample handling. Thus, it is critical to define the differentiation of lipidomic profiles caused by disease as disease-specific changes. Clinic lipidomics needs a good operation protocol with internationalized standards, which should be one of MUST requests in order to ensure the quality of clinic lipidomics data. Analysis methods for biological information of clinical lipidomics are also the challenge to be standardized in the application of clinical lipidomics. Although more diagnostic biomarkers and potential therapeutic targets are consistently discovered and identified for certain diseases, it is still challenging to identify and develop lipid-based and disease-specific biomarkers, and to prevent the pathological processes by alterations in those selected lipid-based biomarkers and target molecules. In understanding the mechanisms of systemic metabolism, the regulation of lipid-specific enzymes should deserve more attentions.

In conclusion, clinical lipidomics is a new extension of lipidomics to study lipid profiles, pathways, and networks by characterizing and quantifying the complete profile of lipid molecules in cells, biopsies, and body fluids of patients. The application of clinical lipidomics is also an emerging science with an unexpectedly increasing speed, although there are more challenges than we expected and no biomarkers are discovered using clinical lipidomics yet. It has more values if lipidomics can be integrated with data of clinical proteomics, genomics, and phenomics. Several challenges, e.g., instability, specificity, and sensitivity, are faced and overcome before clinical application. The association of lipidomic profiles with gene expression and sequencing of lipid-specific proteins/enzymes should be furthermore clarified. Thus, clinical lipidomics is expected to be more standardized during handling and in measurements, sensitive in response to changes, specific for diseases, and efficient in data analyses, to meet clinical needs.

## References

[CR1] Han X (2016). Lipidomics for studying metabolism. Nat Rev Endocrinol.

[CR2] Kim WS, Jary E, Pickford R, He Y, Ahmed RM, Piguet O (2018). Lipidomics analysis of behavioral variant frontotemporal dementia: a scope for biomarker development. Front Neurol.

[CR3] Lv J, Zhang L, Yan F, Wang X (2018). Clinical lipidomics: a new way to diagnose human diseases. Clin Transl Med.

[CR4] Lv J, Gao D, Zhang Y, Wu D, Shen L, Wang X. Heterogeneity of lipidomic profiles among lung cancer subtypes of patients. J Cell Mol Med. 2018. 10.1111/JCMM.13782. 10.1111/jcmm.13782 PMC6156354 29999584

[CR5] Postle AD (2012). Lipidomics. Curr Opin Clin Nutr Metab Care.

[CR6] Vaz FM, Pras-Raves M, Bootsma AH, van Kampen AH (2015). Principles and practice of lipidomics. J Inherit Metab Dis.

[CR7] Wang X (2016). New biomarkers and therapeutics can be discovered during COPD-lung cancer transition. Cell Biol Toxicol.

[CR8] Wang X (2018). Clinical trans-omics: an integration of clinical phenomes with molecular multiomics. Cell Biol Toxicol.

[CR9] Xu M, Wang X (2017). Critical roles of mucin-1 in sensitivity of lung cancer cells to tumor necrosis factor-alpha and dexamethasone. Cell Biol Toxicol.

[CR10] Yang K, Han X (2016). Lipidomics: techniques, applications, and outcomes related to biomedical sciences. Trends Biochem Sci.

[CR11] Yang L, Li M, Shan Y, Shen S, Bai Y, Liu H (2016). Recent advances in lipidomics for disease research. J Sep Sci.

[CR12] Zhu Z, Qiu S, Shao K, Hou Y. Progress and challenges of sequencing and analyzing circulating tumor cells. Cell Biol Toxicol. 2017; 22. 10.1007/s10565-017-9418-5 PMC6132989 29168077

